# Mitigating Cybersickness in Virtual Reality Systems through Foveated Depth-of-Field Blur

**DOI:** 10.3390/s21124006

**Published:** 2021-06-10

**Authors:** Razeen Hussain, Manuela Chessa, Fabio Solari

**Affiliations:** Department of Informatics, Bioengineering, Robotics and Systems Engineering, University of Genoa, 16126 Genoa, Italy; manuela.chessa@unige.it (M.C.); fabio.solari@unige.it (F.S.)

**Keywords:** cycbersickness, spatial blur, depth-of-field, foveation, gaze-contingent, eye-tracker, shader

## Abstract

Cybersickness is one of the major roadblocks in the widespread adoption of mixed reality devices. Prolonged exposure to these devices, especially virtual reality devices, can cause users to feel discomfort and nausea, spoiling the immersive experience. Incorporating spatial blur in stereoscopic 3D stimuli has shown to reduce cybersickness. In this paper, we develop a technique to incorporate spatial blur in VR systems inspired by the human physiological system. The technique makes use of concepts from foveated imaging and depth-of-field. The developed technique can be applied to any eye tracker equipped VR system as a post-processing step to provide an artifact-free scene. We verify the usefulness of the proposed system by conducting a user study on cybersickness evaluation. We used a custom-built rollercoaster VR environment developed in Unity and an HTC Vive Pro Eye headset to interact with the user. A Simulator Sickness Questionnaire was used to measure the induced sickness while gaze and heart rate data were recorded for quantitative analysis. The experimental analysis highlighted the aptness of our foveated depth-of-field effect in reducing cybersickness in virtual environments by reducing the sickness scores by approximately 66%.

## 1. Introduction

The introduction of modern head-mounted displays (HMDs) such as the Oculus Rift and HTC Vive has seen major advancements in the field of virtual reality (VR). These devices facilitate new and novel experiences for users above and beyond what is possible with traditional audiovisual displays. However, its widespread usage has been hindered due to the fact that users tend to feel discomfort after its prolonged usage. This discomfort, which can be referred to as simulator sickness (SS) or eyestrain or visual fatigue, occurs due to the differences in the visual experience between the real world and the virtual world [1]. In VR devices, the virtual environment is presented in pin-sharp focus with the aim of allowing users to extract information from all areas of the projected images [2]. On the contrary, humans focus on objects in their surroundings by continuously altering their eye position and accommodation. Objects located at the accommodative distance form a sharp image on the retinae while other objects appear blurred [3].

Several user studies involving modern consumer-oriented HMDs have been conducted to try to identify different factors that may influence the level of the induced simulator sickness. Occlusion of the external environment (i.e., environment was not displayed) in the virtual world can cause the users to suffer from SS even more [4,5]. An unnatural mapping of senses and simulation errors such as tracking errors and latency can also lead to higher sickness and a lower sense of immersion [6,7]. Users who have prior experience with video games are less susceptible to cybersickness [8]. Personality traits such as neuroticism also have a strong correlation to SS but are mainly related to nausea [9]. Physiological responses to VR stimuli such as pupil dilation, blinking, saccades, and heart rate have been found to have a significant correlation with cybersickness [10]. An increased blink frequency has also been reported among VR users when they suffered from cybersickness [11]. User studies on VR systems have also shown that users are sensitive to artifacts present inside 20° of eccentricity [12].

There are several methods to detect and measure cybersickness [13]. Questionnaires based on self-report responses of users are the earliest methods for assessment and are the de-facto choice for VR systems [14]. There are several types of these questionnaires such as Simulator Sickness Questionnaire (SSQ) [15] and Virtual Reality Sickness Questionnaire (VRSQ) [16]. However, SSQ remains the most popular and most cited among the VR community. Recently, there has been some interest to assess cybersickness through physiological signals such as heart rate, respiratory rate, and skin conductance with promising results [17,18].

Researchers have proposed many techniques to reduce the level of induced cybersickness. Fernandes et al. [19] proposed to dynamically alter the field-of-view depending on the user motion. However, such approaches limit the sense of presence in the virtual world. Alternatively, user studies have been shown to reduce SS by incorporating spatial or defocus blur [20,21]. Budhiraja et al. [22] tried to address sickness caused by vection in VR. Vection is the perception of self-motion in the absence of any physical movement, often caused by secondary moving objects in the user-view. They incorporated rotation blurring, i.e., applying a Gaussian blur to the entire scene when peripheral objects undergo rotational movements. Buhler et al. [23] addressed cybersickness induced from peripheral motion by dividing the scene circularly and reducing optic flow in the peripheral section. Use of vignetting during amplified head movements to counter cybersickness had an opposite effect [24]. Saliency-based dynamic blurring only worked for high speed scenes [25]. Moreover, a recent study demonstrated that introducing spatial blur effects in VR systems can also help with depth perception [26].

In the computer graphics field, depth-of-field (DoF) rendering is a popular approach to incorporate spatial blur. Images are blurred using information from the camera model and the corresponding depth maps. Depth-based spatial blur techniques can be classified into two main categories: object space and image space methods [27]. Object space methods, in order to generate DoF effects, operate directly on the 3D scene and are built into the rendering pipeline. On the contrary, image space methods are considered a post-processing operation since they operate on images and their corresponding depth maps. Object space methods suffer less from artifacts when compared to image space methods. However, image space methods are preferred in VR applications since speed is of utmost importance and image space methods are much faster. In order to avoid artifacts, image space methods need to be tuned carefully. Most commonly encountered artifacts include intensity leakage and depth discontinuity. Intensity leakage is when a blurred background blurs on top of an in-focus object. Depth discontinuity is when the background is in-focus, but the silhouette of the foreground object appears sharp. These artifacts mainly occur when there is an abrupt change in the depth map.

An alternate approach for introducing space variant blur is foveated imaging in which the image resolution varies across the image based on the user’s fixation [28]. Foveated rendering [29,30,31,32] can reduce the computational load for VR devices by providing high acuity to the user’s fixation point and reduced acuity in the peripheral regions. However, foveated rendering provides focus information decoupled from depth cues. A more natural scene can be produced by using a combination of the two [33]. Furthermore, Maiello et al. [34] demonstrated that depth perception can be affected by foveation, and Solari et al. [35] showed that the size and the stimulated portion of the field-of-view can affect perceived visual speed.

In this paper, we develop a system that takes its inspiration from the human physiological system and the optical characteristics of lenses. The proposed system couples the output of foveated rendering and DoF blur to provide an artifact-free scene in the central region. Our system offers smooth transitions when the fixation point changes while providing real-time performance. Current spatial blur techniques applied to VR (discussed in detail in Section 2) often suffer from artifacts or fail to provide sufficient frame rates [36]. Our system provides real-time gaze-contingency for off-the-shelf HMDs that have an integrated eye tracking system using image space methods. We also present a user study we conducted on cybersickness in order to evaluate whether our technique can significantly reduce the level of induced cybersickness in virtual environments. For the user study, we assess cybersickness through the SSQ questionnaire and heart rate measurements.

The paper is organized as follows: Section 2 highlights the related works. Section 3 presents our developed system. The user study designed to evaluate the system is presented in Section 4. In Section 5, we analyze the performance of our system based on the results of the user study. In Section 6, we conclude the paper with a discussion.

## 2. Related Works

In this section, we present some recent works done to introduce space-variant blur in VR systems such as foveated rendering and depth-of-field effects. The aim of this section is to highlight some of the problems faced by the VR community with regard to these topics, which also served as a motivation of our work.

Several attempts have been made to introduce DoF blur effects in VR systems [37,38]. These systems assume a focus distance and use the lens model to compute the circle of confusion. The amount of blur in the peripheral pixels is based on the depth difference between the point of fixation and that particular pixel. However, these systems are not gaze-contingent as they assume either a fixed focus distance or assume the user is always fixated at the center of the scene. Alternatively, gaze contingent systems have also been proposed for near-eye displays [39,40]. These systems use adjustable lenses and can potentially be used to correct hyperopia and myopia in VR systems. The major drawback of such setups is that they are hardware intensive and cannot be adapted to modern lightweight HMDs.

Space-variant resolution can be provided through log-polar mapping [41]. The image is first transformed into the cortical domain and then into the retinal domain to provide an image that has higher resolution in the center and lower resolution as the image coordinates move away from the image center. Such techniques were exploited by Meng et al. [31] who proposed a kernel based foveated rendering approach that maps well to current generation of GPUs. Alternatively, a phase-aligned approach towards foveated rendering has also been developed [42]. Only the high acuity foveal region is aligned with the head movements while the peripheral region is instead aligned with the virtual world. Thus, only the high acuity regions require additional processing in each frame. Current foveated rendering methods use fixed parameters that are often tuned manually. Tursun et al. [32] proposed to use a content aware prediction model based on luminance and contrast to compute the optimal parameters. Lin et al. [43] investigated how the size of the foveal region or the central window influences cybersickness. In their study, they found no correlation between the amount of induced sickness and the size of the central window. However, their study highlighted that users adapt more quickly to larger foveal regions.

A common issue in most foveated rendering techniques is geometric aliasing which appears in the form of temporal flickering and can be easily noticed by users [36]. Some solutions have recently been proposed to overcome these artifacts. Franke et al. proposed temporal foveation built into the rasterization pipeline [44]. They introduce a confidence function based on which they decide whether to re-project the pixels from the previous frame or to redraw them. Their system works relatively well on dynamic objects, which is a bottleneck for most foveated rendering algorithms. Since they do not always use a freshly rendered image as input and rely on data from previous frame to achieve a high computational performance, their system does not work well with reflections and transparent objects. Alternatively, Weier et al. [45] propose adding depth-of-field as a post-step to remove artifacts introduced by foveated rendering algorithms. Their approach showed promising visual results in their user study. However, they were unable to achieve the necessary frame rates to meet the HMD’s V-Sync limit, which is necessary to reduce fatigue and cope with fast eye movements [46]. The authors emphasized the need to combine the DoF blur and foveated imaging to obtain optimal results.

Our proposed technique described in detail in the next section tries to combine the foveation and DoF effects applied to the source image using image space methods which are much faster and support a wider range of VR developing platforms. We use freshly rendered images every frame which allows the system to accommodate a more diverse range of objects in the VR environment. By shifting to image space methods, we are able to meet the V-sync limit of VR HMDs.

## 3. The Proposed Foveated Depth-of-Field Effects

The proposed spatial blur technique incorporates DoF blur and foveation effects. The processing is implemented at the shader level to ensure real-time performance. Image space methods are exploited in the linear color space. Different types of smoothing filters were considered, such as Gaussian filtering, Bokeh [47], and disc effects. However, since the system takes inspiration from the human physiological system, the Bokeh filter was preferred as it better mimics the aperture present in the human eyes and can lead to a more realistic output.

**Figure 1:**
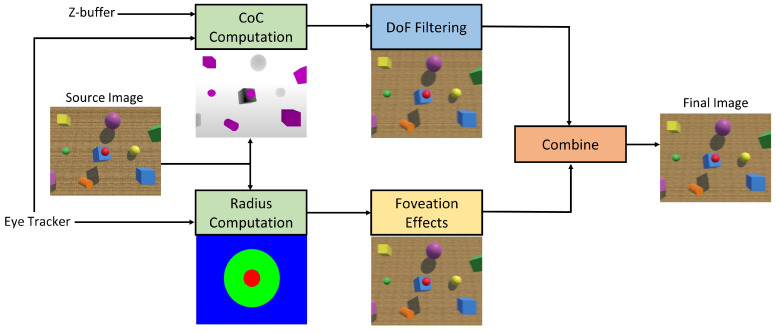
Process flow of the proposed foveated DoF technique showing the intermediate outputs. Fixation is at the center of the red sphere.

The pseudocode of the foveated DoF effects is described in Algorithm 1, while the process flow of the proposed technique is shown in Figure 1. In the first shader pass, the circle of confusion diameters is computed using the raw depth values and stored in a single-channel texture object. The circle of confusion diameters are shown as grey for objects farther from a fixation plane and as purple for objects in between the user and the fixation plane. Simultaneously, the image is divided into three circular sections by computing the distance of each pixel to the fixation pixel. Red pixels represent the pixels in the foveal area, while green and blue pixels represent the near and mid peripheral regions, respectively. Using the source image and the circle of confusion texture, the depth-of-field effects are computed in the second shader pass. Similarly, using the foveation mask and the source image, the foveation effects are computed in the third shader pass. In the last shader pass, the effects are combined to obtain the final output. The smoothing filters are applied at half resolution of the source image and the resultant frames are later up-sampled. Details of the individual processes involved are described in the following subsections.
**Algorithm 1:** Foveated DoF effects for VR
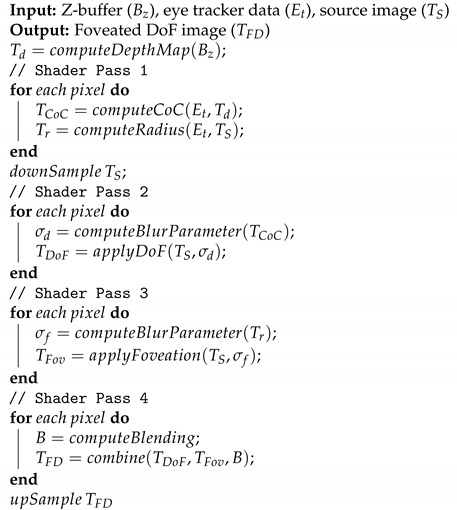


### 3.1. Depth-of-Field Blur

When humans visually perceive their surroundings, the retinal images contain variations in blur. This variation is due to the objects being placed at different depth planes and is an important cue for depth perception. In order to synthesize this blur effect in VR systems, we use a depth texture object to create the depth map of the virtual scene. Depth values corresponding to each pixel on the HMD screen are computed and stored in a Z-buffer. The information inside the Z-buffer is scaled between 0.0 and 1.0 to ensure the system can be used with any HMD configuration. This depth information is used to define the parameters of the smoothing filter. An eye tracker is used to identify the fixation plane and the amount of blur is varied based on the difference in pixel depths (i.e., on the difference in depth of the scene objects with respect to the fixation plane). Objects on the accommodative plane are kept as they are in the source image while a smoothing filter is applied on every other region.

**Figure 2:**
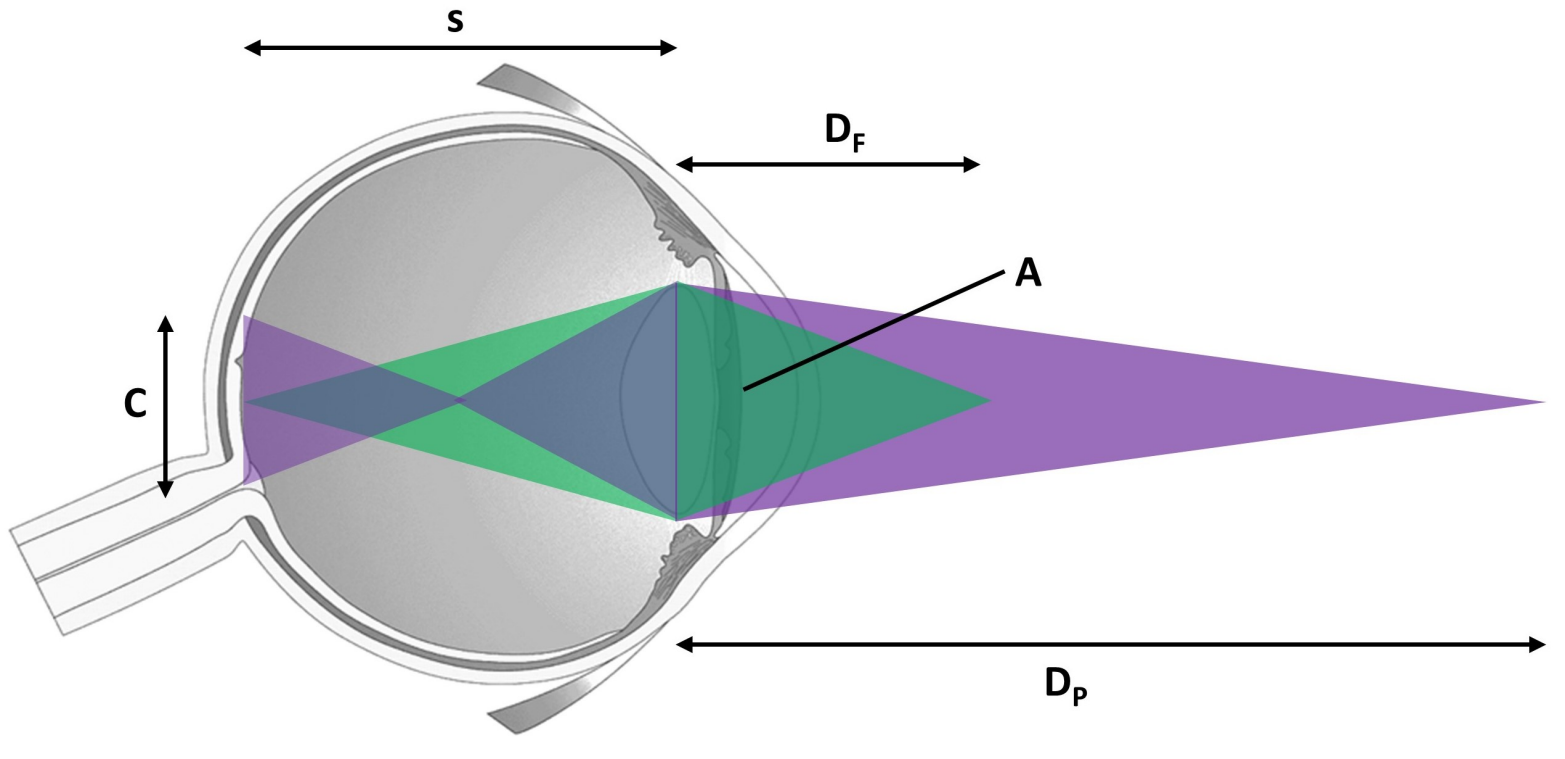
Illustration of the circle of confusion concept. Point of fixation is at distance $D_{f}$. Point located at distance $D_{p}$ forms a circle on the retina with diameter *C*. *A* denotes the aperture and *s* is the posterior nodal distance.

We use the circle of confusion concept from the field of optics to model the amount of blur associated with each pixel. An illustration of the concept can be seen in Figure 2. When the lens is focused at the object placed at distance $D_{f}$, a circle with diameter *C* is imaged on the retina by the object place at distance $D_{p}$. This circle is referred to as the circle of confusion. We use the formulation developed by Held et al. [48] for computing *C* and this is defined by (1):

(1) $$C=As\left|\frac{1}{D_{f}}-\frac{1}{D_{p}}\right|$$

where *s* is the distance between the retina and lens, more commonly known as the posterior nodal distance, and *A* is the aperture of the eye.

We use the circle of confusion to alter the blur associated with each pixel. The bigger the size of *C*, the higher the amount of blur that is present. This implies that the parameter of the blur $\sigma_{d}$ has a direct relation to the size of the circle of confusion, i.e., $\sigma_{d}\propto C$. We adapt (1) to our system and formulate (2) to compute $\sigma_{d}$:

(2) $$\sigma_{d}=K\left|\frac{1}{Z_{f}}-\frac{1}{Z_{p}}\right|$$

where $Z_{f}$ is the depth of the fixation point, $Z_{p}$ is the depth of the rendered pixel, and parameter *K* is the fitting of As and the constant relating *C* and $\sigma_{d}$. The parameter *K* is scene and user dependent and has to be tuned accordingly. We tune this parameter based on the quality index of the image proposed by Wang and Bovik [49]. Image degradation such as contrast loss is often associated with blurred images [32]. We chose the value of K that ensures a sufficient quality index.

**Figure 3:**
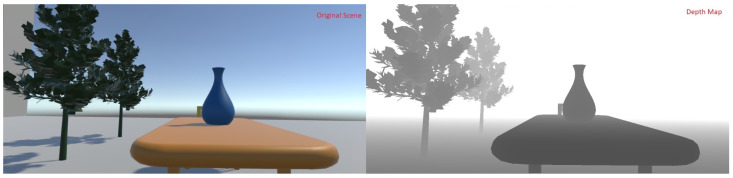
An example scene along with its associated depth map.

**Figure 4:**
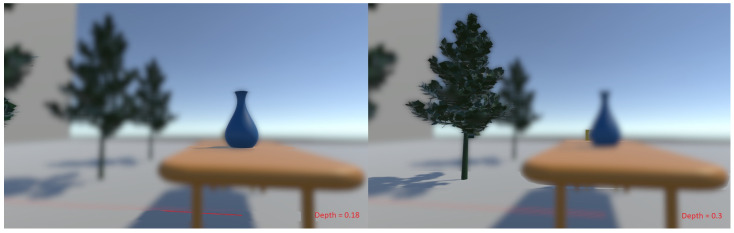
Depth-of-field effects for different planes of fixation. Points of fixation (depth values are reported in red on the images) are on the vase and the front tree in the left and right images, respectively.

A detailed illustration of this depth-of-field effect can be seen in Figure 3 and Figure 4. Figure 3 shows the original scene along with its calculated depth map. Figure 4 shows the output for the plane of fixation at different depths. The plane of fixation in the left image is on the vase. Pixels at the vase depth plane appear sharp. The right image shows the output when the plane of fixation is on the tree. It can be seen that the chair (only partially visible as it is occluded by the vase) also forms a sharp image as it is at the same depth as the tree.

### 3.2. Multi-Region Foveation

**Figure 5:**
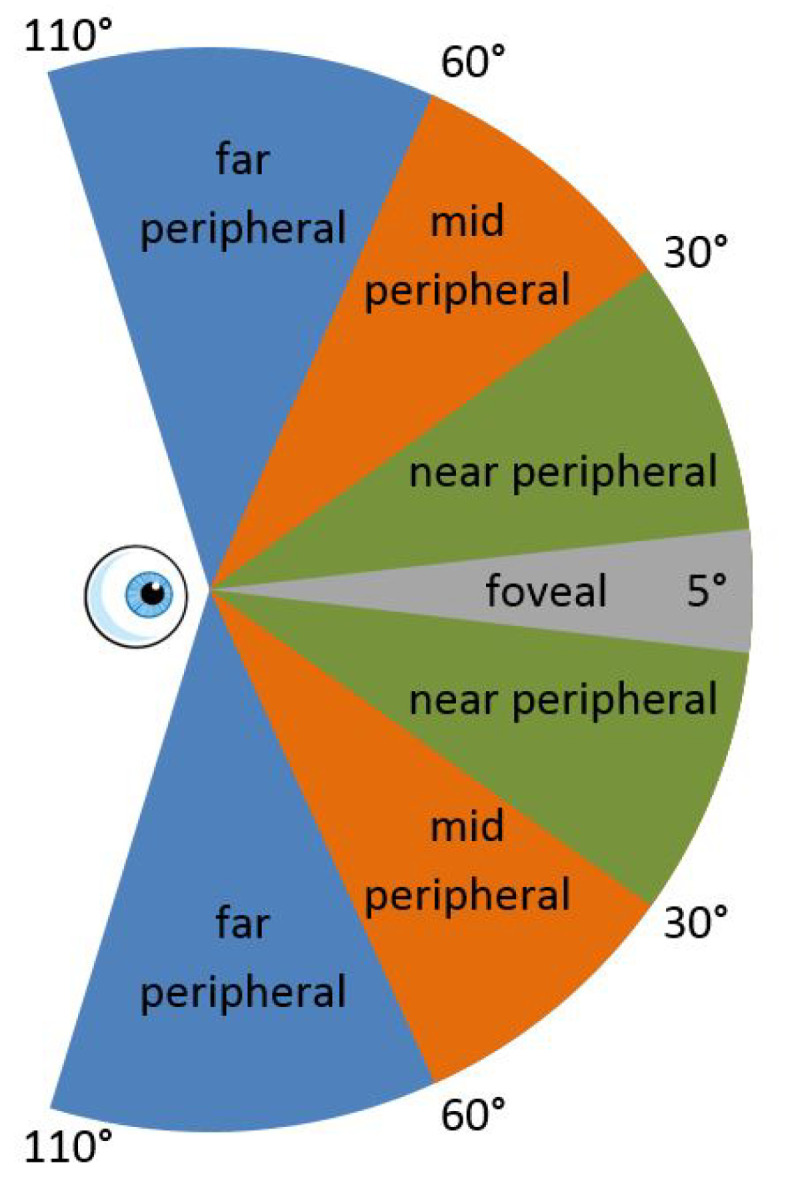
Human field-of-view for both eyes showing the foveal, near, mid, and far peripheral regions.

Human visual field-of-view is composed of foveal and peripheral regions [50]. The divisions of the human visual system can be seen in Figure 5. The central foveal region is sharp and detailed since the light rays entering the eye form a sharp image on the retinae while the peripheral region lacks fidelity and appears blurred on the retinal image due to the decrease in density of the light sensitive cells in the periphery. The peripheral region can be subdivided into three further categories, namely the near, mid, and far peripheral regions. The amount of perceived detail in each region decreases as it moves further from the center. A far peripheral region is only visible to one eye and does not contribute to stereoscopic vision.

We divide the overall imaged scene into three sections corresponding to the foveal, near, and mid peripheral regions. A far peripheral region is not visible in modern HMDs due to their optical limitations and thus is not considered in our system. However, the system can be adapted to include it as well by simply increasing the divisions of the rendered scene. We use circular divisions as opposed to rectangular ones since it better represents the shape of the lenses present in commercially available HMDs. The fixation point is considered the reference center of the circular regions, and the regions are sketched around it. The central division defines the foveal region and is output without any further processing while the smoothing filter is applied to the other regions. The parameter of the blur $\sigma_{f}$ associated with each pixel is determined by the location of that particular pixel in the divided scene. In our implementation, we keep $\sigma_{f_{m}}$ for the mid peripheral region as double the $\sigma_{f_{n}}$ of the near peripheral region.

**Figure 6:**
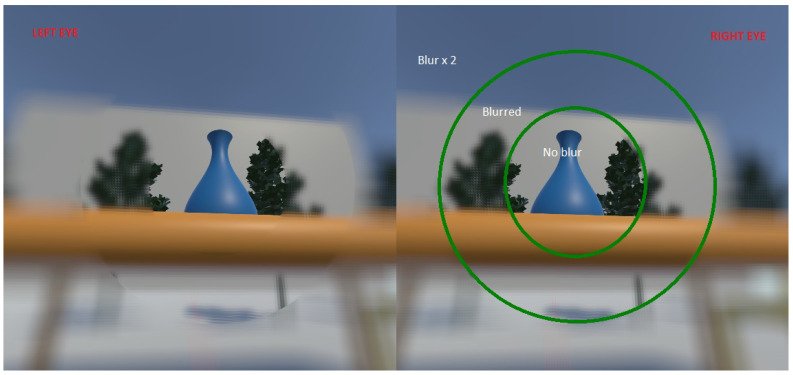
Stereoscopic view of the multi-region foveation output. The central region has no blur applied while the other two regions (highlighted in green for sake of visualization only) have different blurs applied to them.

A detailed illustration of the effect is shown in Figure 6. The middle of the image is assumed as the fixation point in this particular example. From the left eye view in Figure 6, it can be seen that the circular outlines are quite distinct and cause artifacts in the view which can be uncomfortable for the user in its current form.

### 3.3. Artifact Removal and Image Merging

From Figure 4 and Figure 6, it can be observed that some artifacts exist in the resulting images where there is an abrupt change in the blur σ parameter. In order to eliminate/minimize them, we use a technique proposed by Perry and Geisler [51] for blending multi-resolution images using the transfer function of the resolution map. We adapted their approach to our VR system on the transitional regions (i.e., regions with abrupt σ variations). In our system, we use the radial distances between the transitional regions from the fixation point instead of the transfer function.

We introduce the transitional region $R_{t}$ and define the surrounding regions as either inner $R_{i}$ or outer $R_{o}$ based on the location with respect to the fixation point. Likewise, their corresponding radii to the fixation point are defined as $r_{j}$ with j=1,2,3 and $r_{j}<r_{j-1}$. We compute the blending function B(x,y) by (3):

(3) $$B_{j}\left(x,y\right)=\left\{\begin{array}{cc} 0 & d\left(x,y\right)\leq r_{j}\\ \frac{d\left(x,y\right)-r_{j}}{r_{j-1}-r_{j}} & r_{j}<d\left(x,y\right)<r_{j-1}\\ 1 & d\left(x,y\right)\geq r_{j-1} \end{array}\right.$$

where d(x,y) is the distance between the rendered pixel coordinates and the pixel coordinates of the point of fixation.

The output of (3) approaches 1.0 as the considered pixel nears the outer region and approaches 0.0 as the pixel nears the inner region. Using the blending function, we determine the output of the smoothing filter by (4):

(4) $$O\left(x,y\right)=B_{j}\left(x,y\right)I_{j}\left(x,y\right)+\left(1-B_{j}\left(x,y\right)\right)I_{j-1}\left(x,y\right)$$

where $I_{j}\left(x,y\right)$ and $I_{j-1}\left(x,y\right)$ are the outputs from the smoothing filters from *j*th and (j−1)th regions. This makes sure that a percentage from each blur level is taken based on the location of the pixel in the transitional region to determine the final output resulting in an artifact free scene.

**Figure 7:**
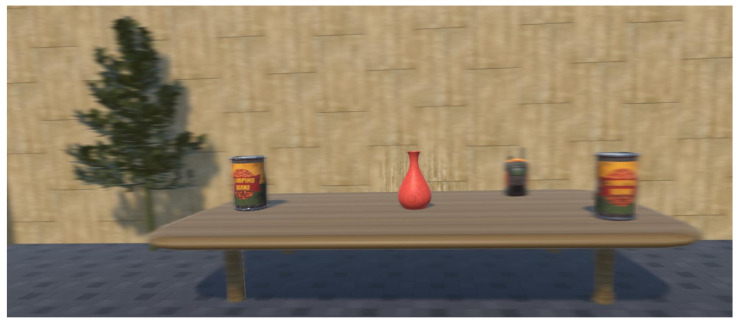
Example of an output from the foveated depth-of-field blur filter.

To merge the output of the DoF blur and foveation, we compute pixel-wise σ for both. However, we only use the smaller σ for the smoothing filter. Figure 7 shows an example output of the foveated DoF effect. The transitions between high acuity and blurred regions are smoother and the central 20° of eccentricity is free of artifacts.

## 4. User Study on Cybersickness

In order to analyze whether the developed foveated DoF effects could help reduce SS while using a VR device, we conducted a cybersickness study. The objective of this study was to measure the level of sickness induced and gather user data for further analysis.

### 4.1. Participants

We collected data from 18 volunteers (9 males and 9 females) aged from 18 to 46 years (mean 29.3 ± 7.6). The participants were volunteers and received no reward. All users had normal to corrected-to-normal acuity and normal stereo vision. All users except four were novice VR users.

### 4.2. Setup

The developed system was implemented using Unity operating on an Intel Core i7-9700K processor equipped with a NVIDIA GeForce GTX 1080 graphics card. An HTC Vive Pro Eye device that has an integrated Tobii eye tracking system was used for interacting with the user. The HMD has a resolution of 1440 × 1600 pixels per eye and a 110° field-of-view. The Scosche Rhythm armband monitor was used to measure the user’s heart rate.

### 4.3. Design

**Figure 8:**
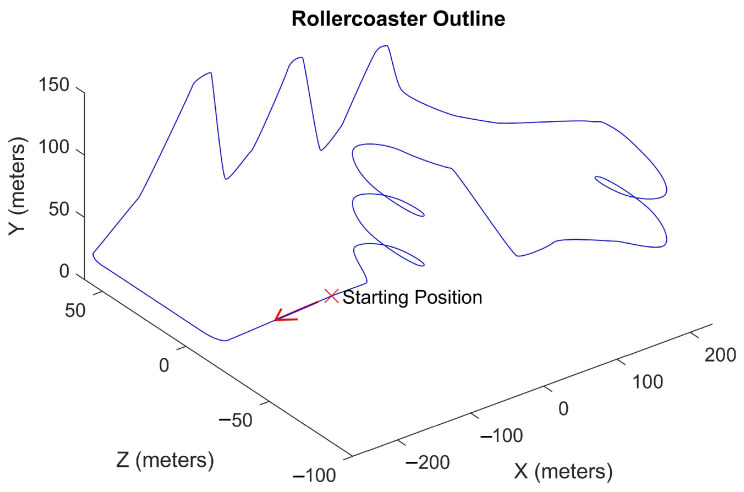
Rollercoaster track outline. The arrow indicates the direction of motion. The coordinate system follows the convention used in Unity, i.e., X: right direction; Y: up direction; Z: forward direction.

**Figure 9:**
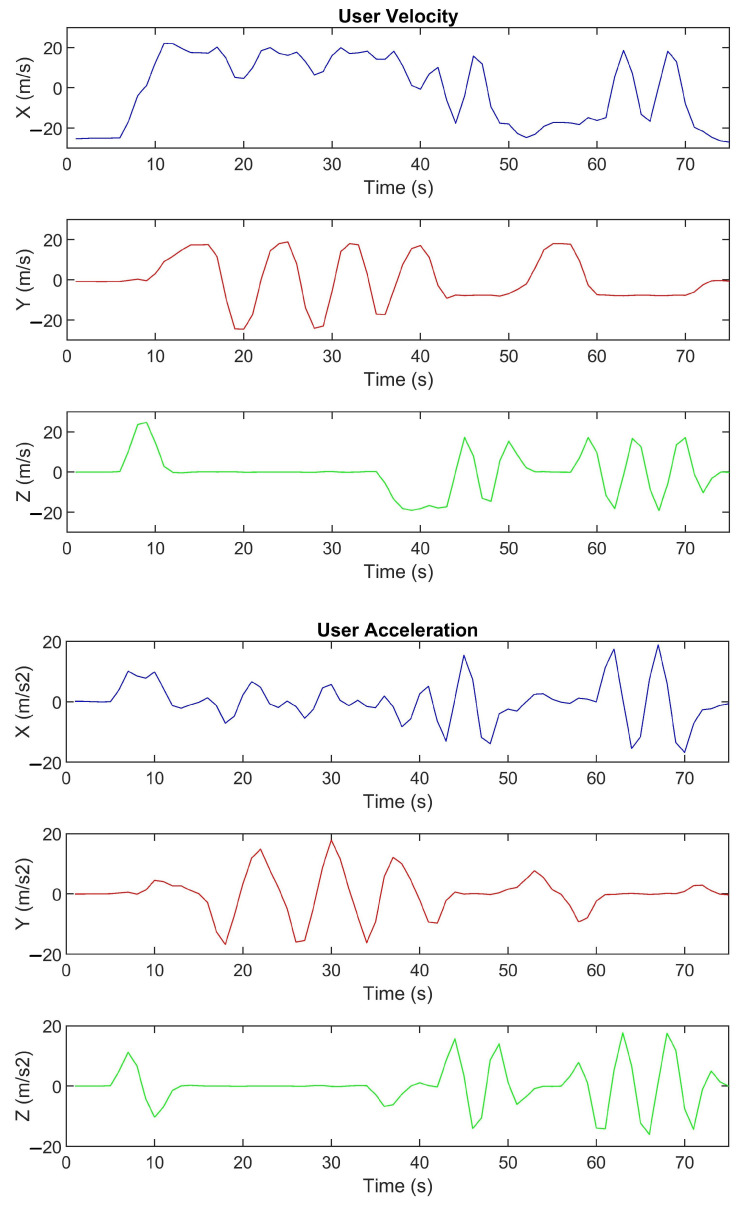
Instantaneous user velocity and acceleration components during each rollercoaster cycle. The coordinate system follows the convention used in Unity, i.e., X: right direction; Y: up direction; Z: forward direction. Seesaw motion: 8–32 s; spiral motion: 36–44 s and 48–64 s.

**Figure 10:**
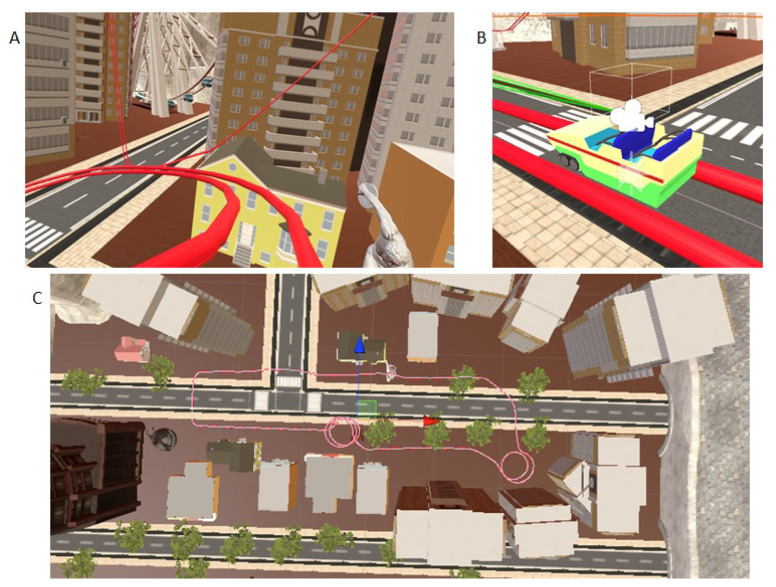
Rollercoaster virtual environment. (**A**) user-view; (**B**) rollercoaster cart with VR camera attached; (**C**) top view of the clustered environment.

A VR rollercoaster environment was designed to induce motion sickness. The rollercoaster was custom-built in Unity in order to have a system which allows us to control and manipulate the experimental parameters, such as velocities, acceleration, and duration of the experiment. The track consists of seesaw and spiral motions placed at different points (see Figure 8). Figure 9 shows the cart velocity and acceleration components over a rollercoaster cycle. Various objects and buildings were closely placed around the rollercoaster tracks to have a clustered environment. The clustered environment ensures that the user’s focus point changes rapidly and the effect of the foveated DoF blur is more prominent. Figure 10 shows the custom VR environment created for the experiment.

### 4.4. Procedure

We consider three conditions: one with our foveated DoF technique enabled (referred to as FD), and one with the Unity’s post-processing stack blur (see the developer’s website https://docs.unity3d.com/Packages/com.unity.postprocessing@3.1/manual/Depth-of-Field.html (accessed on 6 April 2021)) enabled (referred to as GC), and one with the scene with no blur present (referred to as NB). The full fidelity NB condition acts as the control group. The Unity blur GC condition only implements the depth-of-field effect using a 7-pass shader. It also uses the Bokeh effect to introduce spatial blur in the peripheral regions. The size of the Bokeh filter in the Unity blur condition and our foveated depth-of-field condition were kept the same to ensure comparability. The Unity blur does not explicitly support eye-tracking or VR devices so a custom interface was developed to integrate the eye-tracking module with the Unity blur effect to provide gaze-contingency.

All users underwent these three conditions in random order, i.e., 1/3rd of the users performed the FD session first, 1/3rd of the users performed the GC session first, and 1/3rd of the users performed the NB session first. This was to ensure that no bias was present in the experiment. Each session only had one condition active. A significant amount of time was provided between each session to all users to recover from the after-effects of the previous condition. Participants were provided with a minimum of a 90-min break between the sessions. Most users opted to undergo the sessions on successive days. Before each session, the participants underwent an eye calibration process.

Each user session lasted for 5 min. This length of the experimental session was determined based on pre-testing trials which suggested that this time-frame was sufficient to induce SS based on the rollercoaster design. For quantitative evaluation, the user’s positional data, gaze data, and heart rate were recorded. Heart rate data were recorded at 1 Hz frequency while all other data were recorded at approximately 50 Hz frequency.

### 4.5. Analysis

To measure SS, users had to fill the Simulator Sickness Questionnaire (SSQ) [15]. The SSQ consists of 16 questions, to be answered on a 4-point Likert scale. The SSQ scores reflect the level of nausea, oculomotor disturbance, disorientation, and overall severeness of induced sickness. The questionnaire was filled by each user immediately before (Pre) and after (Post) each session. To measure user experience between each type of session, the Igroup Presence Questionnaire (IPQ) [52] was used. The IPQ consists of 14 questions, to be answered on a 7-point Likert scale. Each user filled the IPQ after each session.

## 5. Experimental Results

Data gathered from the experimental sessions were analyzed to have a better understanding of performance of the developed system. Data analysis is described in the following subsections.

### 5.1. Cybersickness and Presence Evaluation

**Figure 11:**
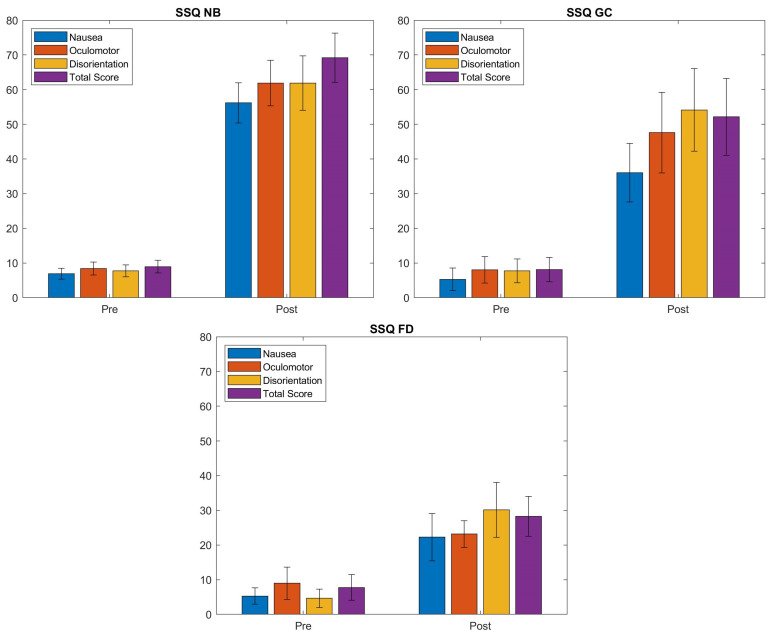
SSQ scores for the cybersickness experiment (conditions: NB—No Blur; GC—Unity Blur; FD—Ours). The questionnaire was filled before (Pre) and after (Post) each session. Each plot shows the mean values, averaged over all the participants, and the standard deviations for the three sub-scales and the overall score.

**Figure 12:**
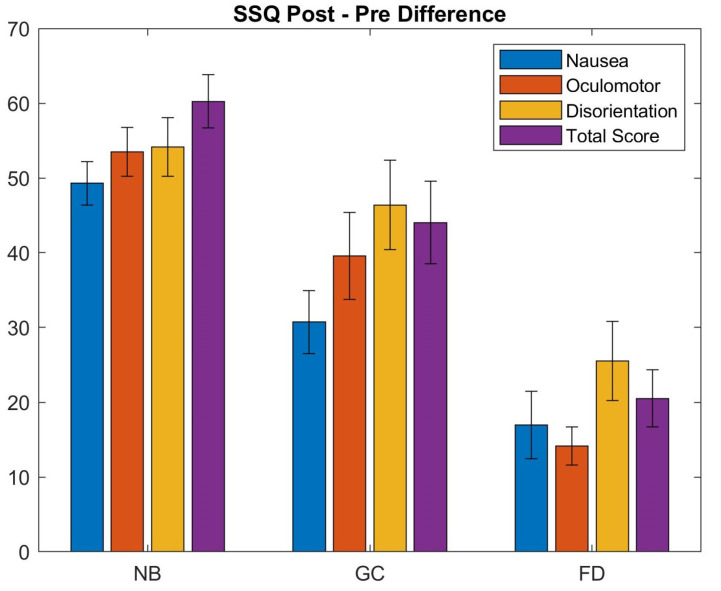
Comparison of the Post-Pre difference of the SSQ scores for each condition (conditions: NB—No Blur; GC—Unity Blur; FD—Ours). The plot shows the changes in individual SSQ scores between the pre and post experiment conditions.

Figure 11 and Figure 12 show the results of the SSQ questionnaire. It can be observed that our foveated DOF blur has a better performance over the no blur setup. A Wilcoxon rank sum test was performed to compare results of the different conditions (see Figure 11). The cross-validation among the pre states of the users who used different blurred systems showed no significant difference between them. The cross-validation between the Pre and Post states of users during each type of system shows a significant difference, i.e., the experimental environment caused a significant increase in the SSQ scores (see Table 1).

The differences between the Pre and Post scores (see Figure 12 and Table 2) show that the amount of increase in individual subscales is highest in NB sessions ranging between 49–54. The conditions with spatial blur incorporated (GC and FD) show the highest change in disorientation scores which is related to the vestibular disturbances. The amount of induced disorientation is similar in the NB and GC conditions. Although the range of individual subscores is different, the results demonstrate that the three conditions produce slightly different patterns of symptomatology, i.e., NB: D ≈ O ≈ N; GC: D > O > N; FD: D > O ≈ N.

Table 3 shows a comparison between different techniques discussed earlier with our foveated DoF effects. We use the difference in the sickness scores between the no effect or full fidelity condition and the best performing parameters for each respective technique. The reported mean SSQ total scores were used where available. One of the user studies did not use the SSQ for the sickness evaluation. The study on peripheral visual effects [23] used a custom questionnaire instead. It can be observed that our foveated DoF blur approach outperforms the other methods.

**Figure 13:**
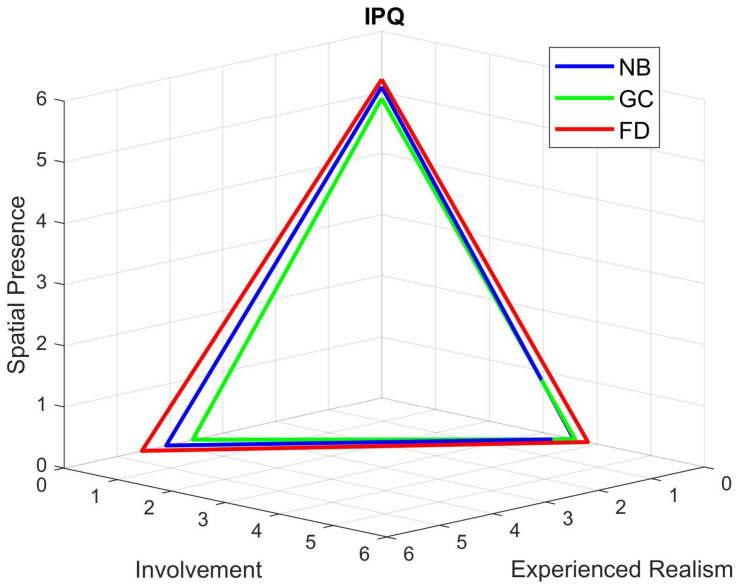
IPQ scores for the cybersickness experiment (conditions: NB—No Blur; GC—Unity Blur; FD—Ours). The questionnaire was filled after each session. NB: Involvement 3.57, Experienced Realism 4.07, Spatial Presence 5.09; GC: Involvement 3.60, Experienced Realism 3.57, Spatial Presence 4.90; FD: Involvement 3.83, Experienced Realism 4.53, Spatial Presence 5.21.

Figure 13 shows the results of the IPQ questionnaire. A Wilcoxon rank sum test between the samples for Unity blur and our foveated DOF against the ones from the no blur sessions displayed no significant differences in the perceived sense of presence between the users of each type of session.

### 5.2. Heart Rate Observations

**Figure 14:**
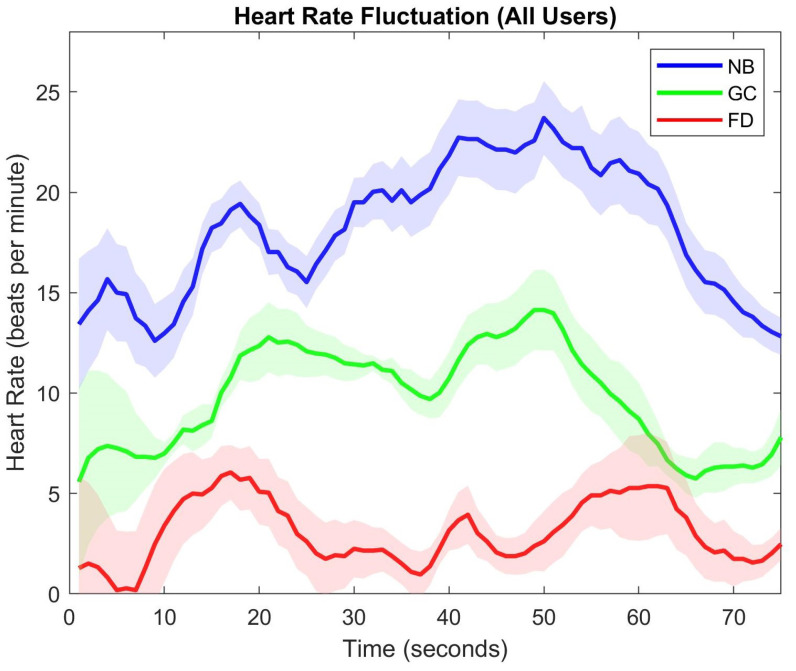
Average heart rate fluctuations from a resting heart rate during a rollercoaster cycle. Origin on the heart rate axis represents the resting heart rate. (conditions: NB—No Blur; GC—Unity Blur; FD—Ours).

Another parameter to observe discomfort is the heart rate fluctuations. However, at the moment, there is no psychophysiological parameter that can satisfactorily measure and predict sickness [53,54], measurements like the finger temperature, reaction time, and heart rate were correlated with cybersickness by Nalivaiko et al. [55]. Figure 14 shows the mean heart rate fluctuations, averaged over all the users, and the standard deviation during a rollercoaster cycle. It can be observed that our foveated DOF blur results in a stable heart rate and only a minute increase from the resting heart rate. On the contrary, the heart rate fluctuation in the no blur sessions is more abrupt. The Unity blur sessions have a median performance. Spatio-temporal data of the user’s movement (see Figure 9) suggest that the spiral/torsional motion has a more adverse effect as compared to seesaw motion (up and down movements). We computed the Pearson’s correlation coefficients between the heart rate fluctuations and the velocity and acceleration data. The results indicate a strong correlation between each other (r-value: NB = 0.87; GC = 0.81; FD = 0.75). It should be noted that the plots in Figure 14 do not begin from the origin because, in each session, there are four rollercoaster cycles, and the plot shows the mean heart rate of the participant, i.e., only in the first cycle, the participants have the resting heart rate while, in the subsequent cycles, there is an aftereffect from previous cycles.

### 5.3. User Gaze Analysis

**Figure 15:**
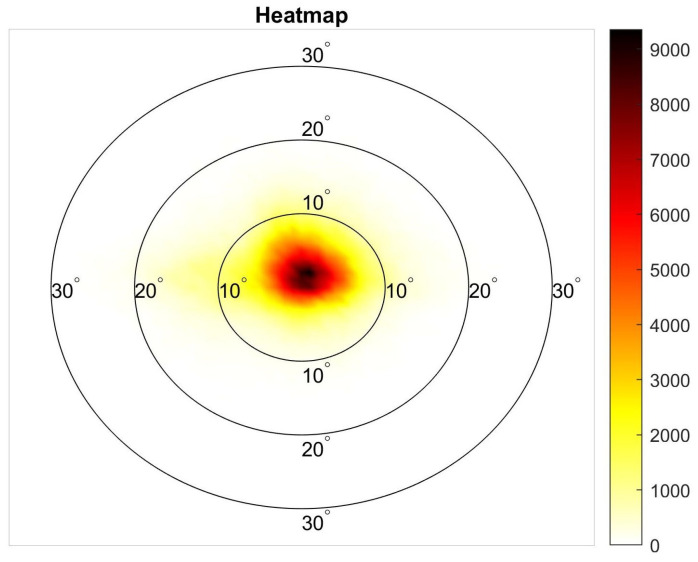
Heatmap of the visual field for user gaze combined for all sessions performed. The circles are centered at the center of the HMD screen and indicate the visual angle (e.g., the 10° circle represents the central 20° of visual eccentricity). The colors represent how frequent the user fixated at that particular location on the HMD screen with white representing 0 and black representing 9358.

In order to better understand how a user behaves/interacts with a VR device, we analyzed the gaze data collected from the experimental sessions. Approximately 4% of the eye tracking data was discarded. This was due to the fact that, during the experiment, for some frames, the users either blinked/closed their eyes or there was faulty sensor reading. Figure 15 shows the combined heatmap of all users. It can be observed that the users tend to fixate mostly on the center of the scene. Positional and orientation data of the user revealed that, when they had to focus on an object further away from the center, they preferred to move their heads instead of just the gaze. This observation is in support of studies conducted by Kenny et al. [56] on first person shooter (FPS) games which highlighted that user gaze is mostly directed towards the center of the view (approximately 85% of the time). Consequently, it can be assumed that gaze related user behavior in VR is similar to FPS games, verifying the assumptions taken in other user studies in the absence of eye tracking [37,38].

We also analyzed the saccadic movements of the users’ eyes. We computed the angular speeds of the eye from the eye tracking data. In humans, angular speed of the eye usually varies between 200°/s to 500°/s, but can go up to 900°/s [57]. Thus, for analysis, we considered mainly the saccades having relatively higher speed ranges to determine whether the motion of the eye has any influence on the induced level of cybersickness. Table 4 describes the peak angular speed measured for each user and how many times speeds of greater than 200°/s was achieved. It can be noticed that, during our blur algorithm integrated sessions, saccades were shorter/slower as compared to the other sessions. A Kolmogorov–Smirnov test was performed on the angular speed data. The statistical analysis showed a significant difference in the distribution of the angular speed data with a 95% confidence interval for the three conditions.

**Figure 16:**
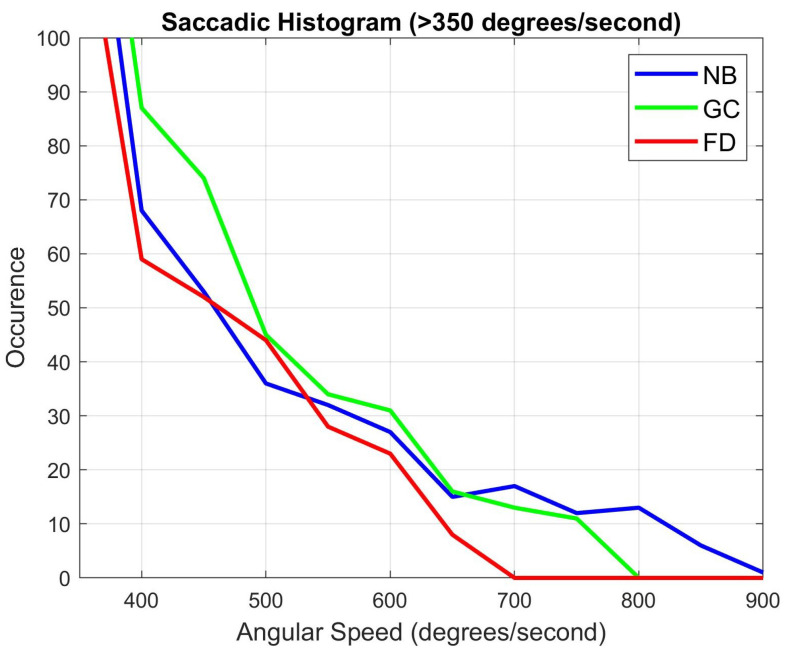
Histogram for angular speed greater than 350°/s of the eye for all users during a saccade.

Figure 16 describes the number of occurrences for speeds higher than 350°/s. It should be noted that speeds lower than this value had a similar trend in all the three conditions, so they are not shown here. Previously, the SSQ revealed that the level of sickness in the no blur sessions is higher than our blur system. Correspondingly, there may be a correlation between the occurrences of faster saccades with the level of induced sickness. The temporal analysis revealed that higher peaks were observed mostly during the seesaw motion.

A possible explanation for lower amplitudes in our system could be that the encompassed blur reduces the amount of detail in the periphery. Consequently, the saccades are shorter. This peripheral reduction mimics the popular approach of reducing the field-of-view to minimize cybersickness [19]. However, in our approach, the peripheral content is still visible, albeit at a lower acuity; thus, the level of presence is not compromised unlike the field-of-view reduction approach.

### 5.4. Age and Gender Variation

**Figure 17:**
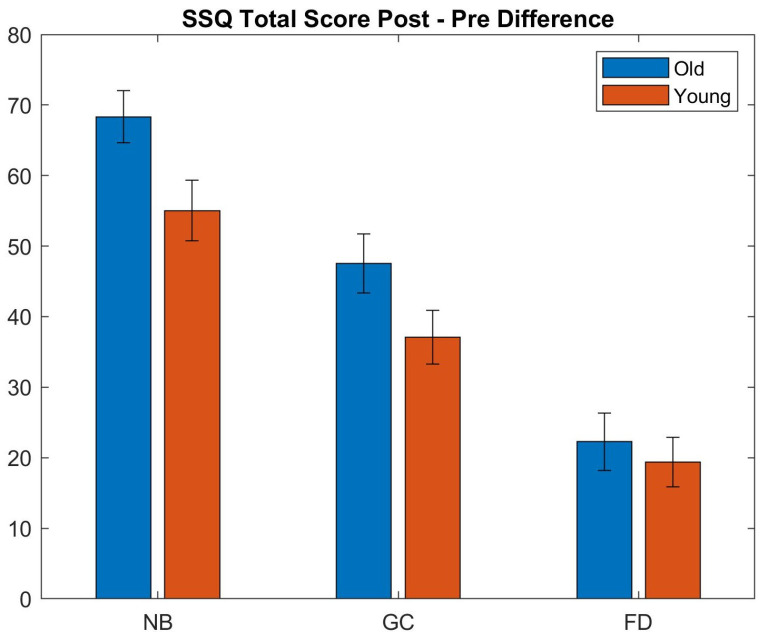
Comparison of the Post–Pre difference of the SSQ scores for each condition with respect to age groups (conditions: NB—No Blur; GC—Unity Blur; FD—Ours). The plot shows the changes in individual SSQ total scores between the Pre and Post experiment conditions for the two age groups. Old: NB 68.34, GC 47.55, FD 22.26; Young: NB 55.03, GC 37.06, FD 19.38.

We also analyzed how age affects cybersickness. It is widely assumed that motion sickness is more prevalent in younger participants; however, past studies on cybersickness in VR have revealed contradicting conclusions. Studies by Arns et al. [58] and Hakkinen et al. [59] revealed that younger participants suffer less from SS, whereas a meta-anlysis by Saredakis et al. [60] showed the opposite. We divided the participants into two groups, young and old. The younger group is comprised of people aged between 18 and 26 years while the rest comprised the older group. There were 10 users in the younger group and eight users in the older group. Figure 17 shows the difference in the total score of SSQ for the two age groups. A Wilcoxon rank sum test was performed. In the FD condition, no statistical difference was found in the SSQ scores and heart rate distributions (*p* > 0.45). However, in the NB and GC conditions, the older participants suffered more from cybersickness (*p* < 0.05).

**Figure 18:**
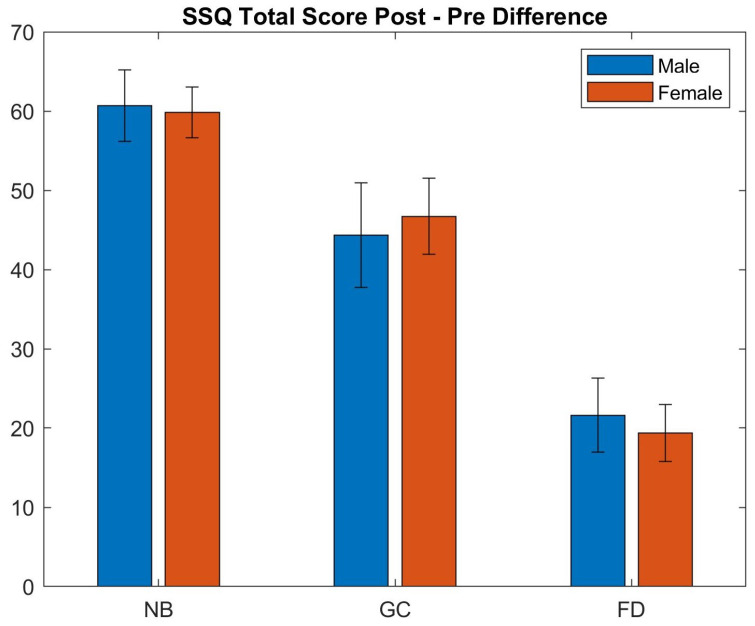
Comparison of the Post–Pre difference of the SSQ scores for each condition with respect to gender groups (conditions: NB—No Blur; GC—Unity Blur; FD—Ours). The plot shows the changes in individual SSQ total scores between the Pre and Post experiment conditions for the two age groups. Male: NB 60.67, GC 44.37, FD 21.63; Female: NB 59.84, GC 46.72, FD 19.39.

The participants were also sub-grouped with respect to gender. Figure 18 shows the difference in the total score of SSQ for the two gender groups. A Wilcoxon rank sum test was also performed; however, no statistically significant difference was found between the two groups (*p* > 0.65). It should be noted that age and gender do not exclusively influence sickness. Factors such as neuroticism, prior VR experience, etc. also simultaneously affect cybersickness. Wider studies on age and gender may be required to fully understand how these factors influence cybersickness as highlighted by Chang et al. [61].

### 5.5. Computational Load Comparison

Using the data recorded from the cybersickness user study, we also calculated the frame processing times in order to have a better understanding of the computational overhead added by the blurring techniques. Data from the no blur sessions acted as the reference for comparison. The average processing times and their equivalent frame rates are summarized in Table 5. There is no overlap between the processing time of the three conditions within a 95% confidence interval. It can be observed that our system offers better computational performance than Unity’s blur even though the built-in blur in Unity only applies the DoF effect, whereas our system processes two different types of blur.

## 6. Conclusions

In this work, we developed a technique for incorporating biologically inspired spatial blur in VR devices with the aim of closing the gap between real world and virtual world experiences. Due to limited field-of-view and near eye displays, modern HMDs provide limited and often mismatching visual cues as compared to the real world. The depth-of-field effect provides an essential cue for depth; however, none of the modern HMDs are able to effectively provide this feature. Foveated imaging is an actively researched field with the aim of reducing the computational load on VR systems by reducing the spatial resolution in the peripheral regions. The technique we developed blends the two blurring procedures to provide a more realistic virtual environment.

The developed system used a Bokeh filter as the main smoothing function. The blurring algorithm used image space methods implemented using a four-pass shader program. Pre-processing was done in the first pass. In the second pass, the DoF blur effects were computed based on the circle of confusion. The third pass divided the VR scene into different circular sections centered around the point of fixation. Each region was assigned a different blur parameter. In the last pass, the outputs from the previous two passes were merged using a blending function to obtain the final rendered scene. The developed system is gaze contingent and offers smooth transitions when the user gaze changes.

We then conducted a user study on cybersickness involving 18 participants. We compared the amount of induced sickness among three types of systems: no blur, Unity post-processing stack depth-based blur, and our foveated depth-of-field blur. A custom-built rollercoaster virtual environment was used to conduct the study. We used the Simulator Sickness Questionnaire to measure cybersickness. For quantitative analysis, we also analyzed heart rate and user gaze measurements. Our analysis showed that there was a statistically significant difference in the level of induced sickness by including spatial blur in the system. There was a 27% and 66% reduction in the SSQ total score for the Unity blur and our technique respectively as compared to the full fidelity condition (mean Post-Pre SSQ score difference: NB = 60.26; GC = 44.05; FD = 20.51). The observations were also supported by the heart rate measurements. It was also observed through the heart rate analysis that circular/spiral motion contributes more adversely to cybersickness as compared to linear motion.

The analysis also showed that older people generally tend to suffer more from cybersickness in immersive VR environments as compared to younger people for the no blur (mean Post-Pre SSQ score difference: old = 68.34; young = 55.03) and Unity blur (mean Post-Pre SSQ score difference: old = 47.55; young = 37.06) conditions. However, there was no statistically significant difference between the two age groups using our blur system (mean Post-Pre SSQ score difference: old = 22.26; young = 19.38). Furthermore, we found no statistical difference in the performance for gender groups.

There are obvious differences between the scenes presented in the three conditions which may help understand why there is lower sickness induced in the systems with spatial blur incorporated. The no blur condition presented the entire VR scene in high focus which contradicts natural viewing. The Unity blur condition mimics how lenses work while our technique considers depth-of-field and foveation effects together as in natural vision leading to a more realistic scene. Another possible explanation to why a reduced sickness is observed is optic flow. Motion in the periphery can cause sickness. Motion is detected by the visual system and hence the motion is seen, but no motion or little motion is sensed by the vestibular system. By reducing the amount of information in the peripheral region, the users are less susceptible to this sensory conflict.

Even though we had a relatively small number of participants, our data indicate that incorporating our blur technique in virtual reality systems can have a soothing effect, potentially decreasing the simulator sickness. As a future work, we will further investigate the resourcefulness of the developed system for mitigating the vergence–accommodation conflict in virtual reality systems. We will also test our system with other virtual reality applications and possibly extend it to augmented reality devices.

## Figures and Tables

**Table 1:** The Wilcoxon rank sum test confidence scores between Pre and Post states for the different subcategories of the SSQ test (N—Nausea; O—Oculomotor; D—Disorientation; TS—Total Score); (conditions: NB—No Blur; GC—Unity Blur; FD—Ours).

	N	O	D	TS
NB	*p* = 0.001	*p* = 0.002	*p* = 0.002	*p* = 0.001
GC	*p* = 0.001	*p* = 0.003	*p* = 0.004	*p* = 0.001
FD	*p* = 0.005	*p* = 0.004	*p* = 0.004	*p* = 0.003

**Table 2:** The mean, standard deviation, and 95% confidence intervals of the Post-Pre difference of the SSQ scores for each condition (N—Nausea; O—Oculomotor; D—Disorientation; TS—Total Score); (conditions: NB—No Blur; GC—Unity Blur; FD—Ours).

	Mean (Standard Deviation)	95% Confidence Interval
NB—N	49.29 (5.81)	[43.14, 55.44]
NB—O	53.48 (6.56)	[46.27, 60.69]
NB—D	54.13 (7.83)	[46.08, 62.19]
NB—TS	60.26 (7.16)	[52.65, 67.85]
GC—N	30.74 (8.44)	[26.91, 34.57]
GC—O	39.58 (11.61)	[33.65, 45.52]
GC—D	46.40 (11.88)	[40.86, 51.94]
GC—TS	44.05 (11.14)	[38.92, 49.17]
FD—N	16.96 (9.07)	[12.97, 20.95]
FD—O	46.40 (5.09)	[10.56, 17.79]
FD—D	25.52 (10.56)	[21.05, 29.99]
FD—TS	20.51 (7.63)	[16.57, 24.42]

**Table 3:** Comparison among different techniques for reducing cybersickness. ΔS is the reduction in the mean sickness scores between the no effect condition and the best performing condition/parameters.

Technique	HMD	VE/Task	ΔS
Dynamic FOV modification [19]	Oculus Rift DK2	Reach waypoints	5.6%
Rotation blurring [22]	Oculus Rift DK2	FPS shooter game	17.9%
Peripheral visual effects [23]	HTC Vive	Find objects	49.1%
FOV reduction (vignetting) [24]	HTC Vive	Follow butterfly	30.1%
Dynamic blurring (saliency) [25]	HTC Vive	Race track	35.2%
Static peripheral blur [43]	HTC Vive Pro	Maze	54.8%
Unity depth blur	HTC Vive Pro Eye	Rollercoaster	26.9%
Foveated DoF (ours)	HTC Vive Pro Eye	Rollercoaster	**66.0**%

**Table 4:** Comparison of angular speed during saccadic motion for each user. Number of occurrences of speeds greater than 200°/s and the peak speed observed are shown. (conditions: NB—No Blur; GC—Unity Blur; FD—Ours).

User	NB		GC		FD	
	>200°/s	Peak	>200°/s	Peak	>200°/s	Peak
AT	106	810°/s	89	502°/s	59	354°/s
CT	132	784°/s	108	544°/s	96	497°/s
EV	88	859°/s	99	743°/s	74	556°/s
GB	136	546°/s	90	650°/s	101	549°/s
HR	115	773°/s	125	663°/s	97	568°/s
KK	78	593°/s	71	539°/s	84	542°/s
LH	132	731°/s	93	707°/s	103	581°/s
MB	87	581°/s	116	582°/s	63	431°/s
MM	112	703°/s	95	697°/s	88	553°/s
ND	101	802°/s	107	718°/s	71	655°/s
NR	86	824°/s	119	702°/s	105	603°/s
OQ	88	595°/s	92	629°/s	95	612°/s
SA	106	697°/s	105	735°/s	94	514°/s
SR	97	710°/s	82	657°/s	68	570°/s
TB	113	688°/s	89	617°/s	87	545°/s
UG	115	591°/s	84	623°/s	89	511°/s
US	92	597°/s	111	502°/s	89	533°/s
YK	67	351°/s	142	661°/s	67	508°/s
Total	1999	859°/s	1923	743°/s	1619	655°/s

**Table 5:** Frame rate comparison (conditions: NB—No Blur; GC—Unity Blur; FD—Ours).

System	Average Processing Time	95% Confidence Interval	Frame Rate
NB	15.9 ms	[15.9 ms, 15.9 ms]	63 Hz
GC	17.2 ms	[17.1 ms, 17.3 ms]	58 Hz
FD	16.7 ms	[16.6 ms, 16.8 ms]	60 Hz

## Data Availability

Not available.
